# Perioperative clinical profiles and longitudinal outcomes in patients supported by continuous-flow left ventricular assist devices: a comprehensive analysis of early and late follow-up data

**DOI:** 10.3389/fcvm.2026.1784700

**Published:** 2026-04-14

**Authors:** Qiuju Ding, Qingqing Zhu, Zeyi Zhou, Cheng Chen, Zhenjun Xu, Jun Pan, Min Ge

**Affiliations:** Department of Cardio-thoracic Surgery, Nanjing Drum Tower Hospital, The Affiliated Hospital of Nanjing University Medical School, Nanjing, China

**Keywords:** follow-up study, heart failure, hospital readmission, survival rate, ventricular assist devices

## Abstract

**Introduction:**

Mechanical circulatory support technology, particularly continuous-flow left ventricular assist devices (CF-LVADs), has emerged as a vital therapeutic option for end-stage heart failure (ESHF). However, domestic data on the long-term efficacy and safety of CF-LVADs in real-world settings remain limited, necessitating further investigation to guide clinical practice.

**Methods:**

This single-center, retrospective study analyzed 28 patients who underwent CF-LVAD implantation as destination therapy for ESHF between October 2022 and July 2025. One patient died intraoperatively due to severe cardiogenic shock and was excluded from subsequent analyses. The remaining 27 patients (median age: 61 years; 88.9% male) were followed for a median duration of 5.4 months (range: 1.1–35.4 months). Perioperative clinical characteristics, early- and late-term outcomes, including survival, readmission rates, and changes in cardiac and renal/hepatic function, were evaluated.

**Results:**

During the follow-up period, significant improvements of liver and renal function were observed, as well as a reduced left ventricular end-diastolic diameter, and an increased left ventricular ejection fraction. Despite these improvements, two deaths occurred during CF-LVAD support, and the hospital readmission rate reached 40.74%.

**Discussion:**

CF-LVADs demonstrated promising efficacy in improving hemodynamic and organ function in patients with ESHF. However, the notable readmission rate and mortality highlight the need for optimized patient selection, postoperative management, and long-term monitoring. This study provides critical insights into the real-world challenges and benefits of CF-LVAD therapy, underscoring its value as destination therapy while calling for larger, multicenter studies to validate these findings and further refine clinical protocols.

## Introduction

Heart failure is a significant public health concern, affecting 1%–2% of the adult population, with an incidence of 100–900 cases per 100,000 individuals annually (1). Patients with end-stage heart failure (ESHF) often exhibit resistance to conventional pharmacological therapies and are usually intolerant of routine surgical interventions due to their poor physical condition. Given its extremely high short-term morbidity and mortality, ESHF not only imposes a substantial health and economic burden on individual patients but also brings immense pressure on the healthcare system (2).

Left ventricular assist devices (LVADs) are critical mechanical circulatory support systems for patients with ESHF, providing partial or complete replacement of left ventricular pump function. Technologically, LVADs have evolved through three generations: (1) Pulsatile-flow devices (e.g., first-generation HeartMate XVE) mimic natural cardiac contraction but are limited by bulkiness and mechanical failure rates, leading to their phase-out (3). (2) Continuous-flow devices (e.g., second-generation HeartMate II) utilize rotary blood pumps with axial or centrifugal mechanisms, offering smaller size and improved durability. However, non-physiological blood flow patterns may increase risks of gastrointestinal bleeding and aortic valve insufficiency (4). (3) Third-generation devices (e.g., HeartMate 3, EVAHEART, Corheart 6) incorporate magnetically or hydrodynamically levitated impellers, reducing shear stress and thrombus formation (5). These devices support physiological pulsatile flow and demonstrate superior survival rates (4). Currently, the application of LVADs primarily applied in three aspects: (1) Bridge to heart transplant (BTT), serving as a temporary measure until a suitable donor heart becomes available. (2) Destination therapy (DT), providing an alternative to heart transplantation for maintaining or alleviating the patient's condition. (3) Bridge to decision (BTD), buying time for clinical decision-making processes.

The continuous-flow left ventricular assist device (CF-LVAD) has emerged as one of the key standard treatment options for patients with ESHF (3). Owing to more precise patient selection, continuously optimized surgical techniques, and increasingly improved perioperative management, the clinical outcomes of patients receiving CF-LVAD therapy have consistently improved (6, 7). Interagency Registry for Mechanically Assisted Circulatory Support (INTERMACS) data revealed that the 1-year and 2-year survival rates of patients treated with these devices were 80% and 70%, respectively (8). These favorable medium-term clinical results have strongly promoted the application of CF-LVAD in the fields of BTT and DT for heart failure. In the United States, the number of CF-LVAD implantations for DT has shown a sharp increase, exceeding 40% of all LVAD implantations by 2012 (8). Moreover, due to the persistent shortage of donor organs, the time patients spend relying on device support while waiting for heart transplantation has been prolonged (9). Additionally, in the actual clinical setting, factors such as blood type incompatibility, size discrepancies, and sensitization necessitate long-term device support in some patients.

China's artificial heart technology had a relatively late, with research in this field gradually initiated after 2000. Currently, four domestically produced third-generation LVAD devices are approved for clinical use: EVAHEART (Chongqing Evaheart Medical Device Co., Ltd., Chongqing, China), CH-VAD (BrioHealth Technologies, Inc., Suzhou, China), HeartCon (Rocor Medical Technology Co., Ltd., Tianjin, China), and Corheart 6 (Shenzhen Core Medical Technology Co., Ltd., Shenzhen, China). These devices incorporate innovations such as the world's smallest full-magnetically levitated pump (Corheart 6, 90 g) and hybrid magnetic-hydrodynamic suspension (HeartCon), overcoming limitations in device size and biocompatibility. By 2022, over 200 LVAD implantations had been performed across 34 Chinese centers. However, the current application of third-generation VADs in China is predominantly concentrated on BTT and BTD, with a notable lack of research and application in the field of DT. Moreover, domestic data on the long-term follow-up assessment of populations with CF-LVAD implantation are currently scarce.

In this study, we therefore, reviewed the our single-center management experience of patients who received CF-LVAD therapy and evaluated the outcomes of this population with early and late follow-up data.

## Methods

### Population and study design

This study was a single-center retrospective analysis that included 28 patients with ESHF who underwent CF-LVAD implantation at our center from October 2022 to July 2025. We excluded patients who had indications for heart transplantation or who could obtain a suitable donor heart within a reasonably foreseeable timeframe under the current medical resource conditions. Consequently, all patients in our study were identified as candidates for destination therapy. The inclusion criteria for this cohort were defined as: (1) meeting the diagnostic criteria for ESHF and intolerance to conventional anti-heart failure drug therapy, (2) aged ≥ 18 years, and (3) provision of a signed informed consent form for LVAD implantation and data use. The exclusion criteria were as follows: (1) aged < 18 years; (2) death within 48 h after LVAD implantation; (3) inability to tolerate conventional anticoagulation or surgery; (4) uncontrolled infections; (5) end-stage diseases of other organ systems, such as malignancies or uremia; and (6) psychological disorders, mental illnesses, or cognitive impairments preventing compliance with treatment instructions.

All patients received standard median sternotomy under general anesthesia and cardiopulmonary bypass support. The LVAD pump was implanted through the left ventricular apex and the outflow graft was anastomosed to the ascending aorta. Postoperatively, the patients received standardized and consistent management strategies from the same Cardiac Surgery Intensive Care Unit (CICU) team according to the established clinical guidelines (10). The perioperative management pathways across different LVADs are fully guided and participated in by engineers from respective manufacturers throughout the process, while maintaining fundamental consistency. The specific measures included: (1) Hemodynamic monitoring: maintaining mean arterial pressure (MAP) around 75–90 mmHg; (2) Anticoagulation strategy: heparin anticoagulation was initiated 6 h after surgery, with a target activated partial thromboplastin time (APTT) of 40–60 s. It was gradually transitioned to warfarin anticoagulation, with a target international normalized ratio (INR) of 2.0–2.5; (3) Infection prevention: prophylactic use of first-generation cephalosporins within 48 h after surgery; (4) Volume management: daily echocardiography was performed to balance the left and right heart chambers and maintain periodic opening of the aortic valve.

This study was approved by the Ethics Committee of Nanjing Drum Tower Hospital (Approval Number: 2025-1290-01). Due to the retrospective nature of the study, the requirement for patients to sign informed consent forms was waived. All patients had signed surgical informed consent forms before surgery, which explicitly included authorization for the use of medical data for clinical research and quality improvement.

### Baseline data collection

We collected and evaluated perioperative clinical profiles, lengths of hospital and CICU stays, and postoperative complications. Specifically, preoperative traits included patients' demographics and clinical characteristics [age, gender, body surface area (BSA), comorbidities, aetiology of heart failure], baseline hemodynamic parameters [MAP, central venous pressure (CVP), pulmonary artery wedge pressure (PAWP), mean pulmonary artery pressure (mPAP)], use of mechanical support [intra-aortic balloon pump (IABP) or mechanical ventilation], and laboratory test results [blood urea nitrogen (BUN), serum creatinine, cystatin C (Cys-C), total bilirubin (TB), alanine aminotransferase (ALT), aspartate aminotransferase (AST), white blood cell count (WBC), platelet count, and INR]. Intraoperative data included blood loss and transfusion requirements, duration of cardiopulmonary bypass, and aortic cross-clamp time. Postoperative monitoring indicators contained hemodynamic parameters, results of the first-day postoperative transesophageal echocardiography assessment [ratio of the left and right heart chambers and inferior vena cava (IVC) width], LVAD pump operating parameters, and fluid intake and output records for the first three postoperative days in the CICU. Early postoperative adverse events included ventricular arrhythmias, bleeding, re-sternotomy, sepsis, stroke, and recurrent heart failure.

### Follow-up and data collection

The regular follow-up was initiated for all patients after their first discharge, with a frequency of once a month, and emergency follow-up was initiated in case of clinical adverse events. The actual follow-up intervals were dynamically adjusted based on the patient's location and medical needs. As of 30 October 2025, when follow-up was completed for all patients, with a median follow-up time of 5.4 months and a maximum follow-up of 35.4 months. The clinical follow-up completion rate was 100%. Late adverse events included death, re-hospitalization, pump-related adverse events (pump malfunction, thrombosis, and infection), major cerebral events (cerebral infarction and cerebral hemorrhage), recurrent heart failure, arrhythmias, non-pump-related infections (pneumonia and poor wound healing), and other causes including mental problems.

To assess terminal organ function after CF-LVAD implantation, biochemical data, including BUN, creatinine, TB, ALT, and AST levels, were collected at 1 month, 3 months, 6 months, 1 year, and 2 years postoperatively. Meanwhile, all available echocardiography reports were retrospectively collected to assess the left ventricular end-diastolic dimension (LVDd), left ventricular ejection fraction (LVEF), and the severity of aortic regurgitation (AR) and tricuspid regurgitation (TR). The severity of AR and TR was classified as none to trace, mild, mild to moderate, moderate, moderate to severe, and severe. AR and TR were considered significant if the grade was moderate or higher. Data were obtained from the structured electronic medical record system of our center.

### Statistical analysis

Continuous variables were assessed for normality using Shapiro–Wilk tests. Normally distributed data are expressed as mean ± standard deviations (SD); non-normally distributed data as median [interquartile ranges (IQR)]. Categorical variables are presented as counts and percentages (*n*, %). Non-parametric comparisons of preoperative and postoperative data were performed using the Wilcoxon rank-sum test. Analyses were conducted using SPSS version 25.0 (IBM SPSS, Inc., Armonk, NY) and GraphPad Prism version 10.0 (GraphPad Software, Inc., La Jolla, CA). A *p*-value < 0.05 was considered statistically significant.

## Results

### Baseline characteristics

During the study period, 28 patients received continuous-flow LVADs, with one patient excluded owing to intraoperative death due to severe cardiogenic shock. The median age was 61 years, and 88.9% of patients were male. The mean BMI was 24.57 ± 3.94 kg/m², and the median BSA was 1.82 (1.67–1.89) m². History of smoking and alcohol consumption were noted in 14 and 5 patients, respectively. Five patients had a history of implantable cardioverter-defibrillator implantation. Coronary artery disease was present in 55.6% of patients; however, only 14.8% underwent percutaneous coronary intervention. Hypertension and diabetes were common comorbidities (48.1% and 55.6%, respectively), with chronic kidney disease (CKD; 11.1%) and atrial fibrillation (29.6%) being less frequent. One patient had stroke-related hemiplegia, and three had ventricular arrhythmias. One patient had a history of prior cardiac surgery. HF was caused by idiopathic cardiomyopathy in 66.7% of patients, the remaining cases were attributed to ischemic etiology.

Most patients (77.8%) were classified as Interagency Registry for Mechanically Assisted Circulatory Support (INTERMACS) levels 1–4, and 48.1% were New York Heart Association (NYHA) functional class IV. Continuous catecholaminergic support was needed in 22.2% of the patients, with one requiring extracorporeal membrane oxygenation (ECMO). Cardiac biomarkers showed elevated brain natriuretic peptide (BNP) or NT-proBNP levels. Echocardiography showed an enlarged left ventricular end-diastolic diameter (LVDd) of 7.24 ± 0.78 cm and borderline right ventricular function, as indicated by a tricuspid annular plane systolic excursion (TAPSE) of 1.57 ± 0.28 cm, an S′ velocity of 8.5 (7.3, 10) cm/s, and a fractional area change (FAC) of 35.3% ± 6.6%. Approximately 44.4% of the patients had severe mitral regurgitation, with severe AR and severe TR each present in one patient.

Regarding pharmacotherapy, 66.7% of patients took mineralocorticoid receptor antagonists and beta-blockers. Approximately 44.4% of patients received vericiguat, with fewer treated with sodium-glucose cotransporter-2 inhibitors (37.0%) and angiotensin receptor-neprilysin inhibitors (29.6%). The baseline characteristics of the study population are summarized in Supplementary Table S1.

### Operative and post-operative managements of patients with CF-LVAD

All patients underwent LVAD as destination therapy. A Corheart 6 (Shenzhen Core Medical Technology Co., Ltd., Shenzhen, China) was implanted in 20 patients, an EVAHEART (Chongqing Evaheart Medical Device Co., Ltd., Chongqing, China) was implanted in 2 patients, and a HeartCon (Rocor Medical Technology Co., Ltd., Tianjin, China) was implanted in 5 patients. Different concomitant procedures were performed in 20 patients during the index operation, and one patient received a right ventricular assist device. The median operative time was 325 (300, 409) min, with average cardiopulmonary bypass and aortic cross-clamp times of 157.31 ± 30.87 and 106.73 ± 23.8 min, respectively. The median blood loss was 1,200 (900, 1,400) mL, and the average blood transfusion volume was 1,380.63 ± 618.84 mL.

Postoperatively, patients were transferred to the CICU, where the median rotational speed of the LVAD was 2,599 (2,498, 2,797) rpm, with an average flow rate of 3.98 ± 0.8 L/min. The median vasoactive inotropic score (VIS) (11) was 53 (33, 85), mean arterial pressure was 78.33 ± 10.02 mmHg, CVP was 13 (12, 16) mmHg, and PAWP was 15 (11, 18) mmHg. Transesophageal echocardiography showed balanced left and right ventricular sizes, with an IVC diameter of 21.3 ± 1.45 mm and a variability of 9 (8, 15) %. The patients received respiratory and circulatory support therapy, with a median duration of inotropic drug use for 5 (3, 7) days and mechanical ventilation time of 17.77 (14.67, 20.30) hours. The median CICU stay was 7 (5, 13) days, and the total hospital stay was 48 (39, 56) days. Operative and postoperative outcomes are summarized in Supplementary Table S2.

### Early and late clinical outcomes of patients on CF-LVAD support

In the early postoperative period, five patients required continuous renal replacement therapy (CRRT) due to acute kidney injury. Eight patients experienced ventricular arrhythmias. One patient underwent re-sternotomy due to cardiac tamponade caused by bleeding. Three patients developed postoperative pulmonary infections, one patient had a bloodstream infection, and one patient experienced poor wound healing. All five recovered after active anti-infective treatment. One patient had a stroke without sequelae. Two patients developed acute left heart failure, which was relieved by adjusting the rotational speed of the LVAD.

**Figure 1:**
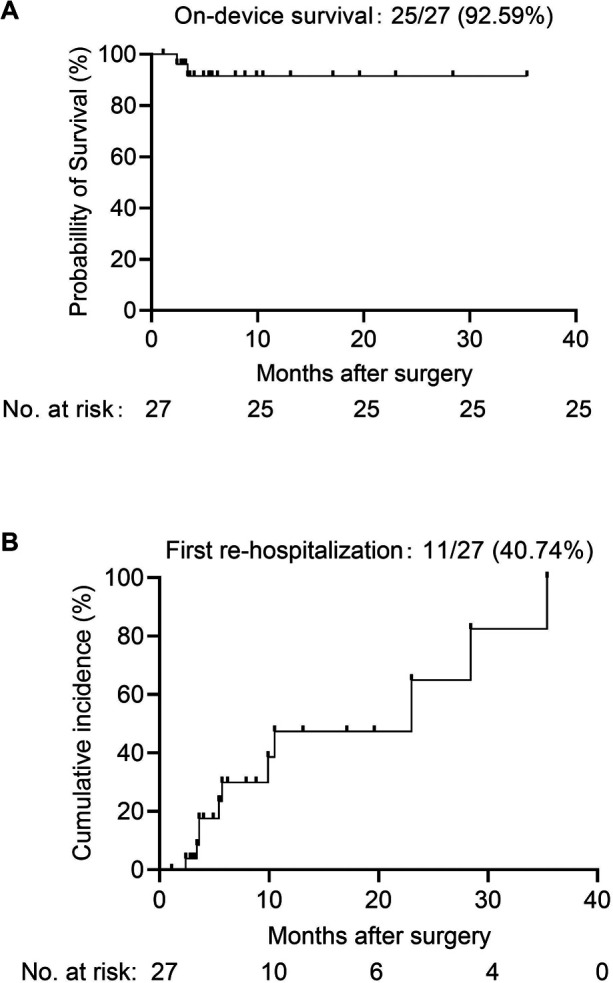
**(A)** On-device survival and **(B)** hospital readmission rate of patients receiving CF-LVAD implantation.

During the follow-up period after discharge, two patients (7.41%) died on LVAD support (one due to brainstem hemorrhage and the other due to septic shock). Kaplan–Meier survival analysis showed that the 1-year device-based survival rate was equivalent to the 3-year device survival rate, both at 92.59% (Figure 1A). Moreover, a total of 11 patients (40.74%) were readmitted to the hospital 19 times due to adverse events. The main reasons for readmission included recurrent heart failure (22.22%), device infection (7.41%), stroke (3.70%), non-device-related infections (7.41%), pleural effusion (11.11%), poor wound healing (3.70%), mental problems (7.41%), and arrhythmias (7.41%). Based on the Kaplan–Meier analysis, the estimated 1-year and 2-year readmission rates were 47.39% and 64.92%, respectively (Figure 1B). The early and late mortality and readmission for adverse event in these patients is summarized in Table 1.

**Table 1:** Early and late mortality and morbidity of the patients on CF-LVAD support.

In-hospital mortality, *n* (%)	
Cardiogenic shock	1 (3.57%)
In-hospital morbidity, *n* (%)	
Cardiac arrhythmia	8 (29.63%)
Reoperation for bleeding	1 (3.70%)
Acute renal failure requiring dialysis	5 (18.52%)
Sepsis	5 (18.52%)
Stroke	1 (3.70%)
Heart failure	2 (7.41%)
Late mortality, *n* (%)	
Stroke	1 (3.70%)
Sepsis	1 (3.70%)
Rehospitalization, no. of events (per patient-y)	
Bleeding	0 (0.00%)
Device thrombus/malfunction	0 (0.00%)
Device infection	2 (7.41%)
Heart failure	6 (22.22%)
Cardiac arrhythmia	2 (7.41%)
Stroke	1 (3.70%)
LVAD nonrelated infection	2 (7.41%)
Pleural effusion	3 (11.11%)
Poor wound healing	1 (3.70%)
Psychiatric issues	2 (7.41%)

In addition, the liver and kidney functions of these patients recovered within 1 month on CF-LVAD support, and these improvements were maintained throughout the entire LVAD support period, although the inter-group comparisons did not reach statistical significance. Moreover, echocardiography records showed that the LVDd of these patients significantly decreased from 7.24 ± 0.78 cm to 5.73 ± 1.17 cm one month after LVAD support (*P* < 0.001) and remained stable thereafter. None of these patients developed new severe valve regurgitation. Table 2 displays the recovery status of organ functions after CF-LVAD implantation.

**Table 2:** Time course of hepatic and renal function during CF-LVAD support.

Variable	Baseline	1 month	3 months	6 months	12 months	24 months	*p*-value
AST (U/L)	34.88 ± 31.41	70.75 ± 51.27	27.61 ± 6.45	30.04 ± 14.59	24.77 ± 6.72	20.65 ± 2.62	0.311
ALT (U/L)	47.25 ± 68.41	50.20 ± 22.34	21.76 ± 9.41	27.74 ± 15.77	22.87 ± 6.43	20.00 ± 12.63	0.702
Total bilirubin (umol/L)	24.81 ± 21.05	17.20 ± 0.28	15.14 ± 6.22	15.36 ± 5.46	15.87 ± 3.62	13.25 ± 4.60	0.471
BUN (mmol/L)	10.84 ± 5.70	9.42 ± 6.24	14.18 ± 10.02	8.63 ± 2.59	7.40 ± 2.64	5.83 ± 1.59	0.206
Creatinine (umol/L)	116.41 ± 83.37	118.50 ± 105.36	118.63 ± 70.74	89.13 ± 25.74	85.83 ± 21.95	83.00 ± 21.83	0.82
Ejection fraction (%)	25.81 ± 5.54	30.77 ± 7.10	30.21 ± 6.22	30.82 ± 6.90	31.54 ± 6.84	35.00 ± 5.29	0.03
LVDd (cm)	7.24 ± 0.78	5.73 ± 1.17***	6.27 ± 0.80*	6.24 ± 0.88*	6.17 ± 1.03*	5.95 ± 0.05*	<0.001

ALT, alanine aminotransferase; AST, aspartate aminotransferase; BUN, blood urea nitrogen; LVDd, left ventricular end diastolic diameter.

* Represented *P* < 0.05.

** Represented *P* < 0.01.

*** Represented *P* < 0.001.

## Discussion

With the widespread application of implantable CF-LVAD technology and the accumulation of extensive management experience, multiple studies have consistently validated the therapeutic efficacy of this intervention in patients with ESHF. Data from the INTERMACS show that the 1-year and 2-year survival rates of patients receiving CF-LVAD therapy reach 80% and 70%, respectively (8). Early clinical trials of HeartMate II as BTT therapy reported an overall 1-year survival rate of 68% (3), while data from long-term follow-up based on the latest cohort indicate that this proportion has significantly increased to 85% (7). The recently conducted ADVANCE BTT trial, which utilized the HeartWare ventricular assist device (HeartWare Inc, Framingham, Mass, USA), showed that the survival rate from device implantation to 180 days after heart transplantation was as high as 94% (12). In the randomized controlled trial of HeartMate II as DT, the 2-year survival rate also improved from 58% in the early cohort to 63% in the later cohort (13). This evidence suggests that CF-LVAD recipients can achieve satisfactory early- and mid-term survival benefits under the current medical system. However, existing studies still lack sufficient long-term data on CF-LVAD support beyond 2 years.

This study systematically summarizes the clinical characteristics of the patients at our center in applying CF-LVAD as the endpoint treatment for ESHF, with a focus on analyzing its early and late outcomes. The results show that LVAD implantation can effectively improve left ventricular systolic function and organ perfusion in patients with ESHF, providing a satisfactory survival rate expected to three years of 92.59%. Investigation into the causes of deaths in the two deceased patients revealed an unmonitored excessive anticoagulation leading to cerebral haemorrhage in one case, while the other case was due to a refractory pulmonary infection caused by multidrug-resistant bacteria (*Klebsiella pneumoniae*), ultimately resulting in septic shock. This study confirms the crucial value of CF-LVAD in the treatment of ESHF, although the management of long-term complications still requires further optimization.

Consistent with the data from the Randomized Evaluation of Mechanical Assistance for the Treatment of Congestive Heart Failure (REMATCH) (14), our data also showed a relatively high readmission rate. The main reasons for readmission in this study were cardiac-related adverse events (heart failure and arrhythmias), infections, and bleeding, which are similar to the major late adverse events reported in the HeartMate II clinical trials, namely arrhythmias, bleeding, and infections. Hashin et al. also reported similar clinical outcomes (15). However, previous reports have indicated higher rates of bleeding and infection, with most cerebrovascular accidents occurring within the first 30 days postoperatively (6). These differences may be attributable to varying anticoagulation management strategies and infection prevention protocols (including regular device tunnel dressing changes and prophylactic antibiotic use) across different centers. Notably, there were no thrombotic events in our study, suggesting that adequate anticoagulation therapy (warfarin and aspirin) combined with novel pump design (magnetic levitation bearing system) significantly reduces the risk of thrombosis. Considering that most CF-LVAD recipients require readmissions frequently, implementing more refined and effective post-discharge management could significantly improve patients' long-term survival rates.

In the early period after LVAD implantation, common complications include infections, bleeding, arrhythmias, heart failure, cerebrovascular accidents, organ dysfunction, and psychiatric issues such as delirium or other cognitive impairments (4). In this study, postoperative arrhythmias were observed in eight patients, predominantly ventricular arrhythmias (including non-sustained ventricular tachycardia and electrical storms), which occurred mainly within the first 30 days after surgery. Previous studies have suggested that ventricular arrhythmias and electrical storms are extremely common following LVAD implantation (16), and the underlying mechanisms include pre-existing ventricular arrhythmias or a history of ventricular tachycardia ablation, use of antiarrhythmic drugs, myocardial injury caused by surgery, and influence of perioperative mechanical circulatory support (17, 18). Although ventricular arrhythmias under LVAD support do not directly lead to hemodynamic collapse, their persistent presence can increase the risk of right ventricular failure. In addition to implantable cardioverter-defibrillator therapy, interventions to minimize the risk of arrhythmia recurrence should be considered, such as the use of antiarrhythmic drugs (e.g., beta-blockers), catheter ablation, or even intraoperative maze procedures (19, 20).

Infection is also one of the common and critical complications in LVAD patients, with an incidence ranging from 19% to 39% (21), leading to high mortality rates when severe. In our cohort, pump-related infections were relatively rare. During the early postoperative period, five patients developed non-device-related infections, with pulmonary infections being the most common. Pulmonary infections encompass pathogen-confirmed pneumonia and clinically-diagnosed infection associated with abnormal inflammatory markers or characteristic imaging findings. Specifically, three patients in this study had mild, non-pathogen-confirmed pulmonary infection. For these patients with evidence of postoperative infection, different levels of antibiotic therapy were administered based on the infection severity, combined with fiberoptic bronchoscopy for sputum aspiration. Additionally, one patient developed incisional infection, which was due to malnutrition. For this patient, we performed debridement and irrigation combined with aggressive anti-infective treatment and nutritional support. All patients were eventually cured. In the late postoperative period, two patients were readmitted due to lead tunnel and device infection, and both improved and were discharged from the hospital. Two other patients were readmitted due to pneumonia. The first patient was discharged improved while the other died from septic shock complicated by refractory pneumonia (*Klebsiella pneumoniae*).

Due to the necessity of device anticoagulation, bleeding is also an unavoidable complication, with possible mechanisms including acquired von Willebrand disease (22), gastrointestinal arteriovenous malformations associated with reduced pulsatility (23), and impaired platelet aggregation (24). In this study, one patient developed pericardial effusion due to bleeding in the early postoperative period and had to undergo a second sternotomy; another patient died from brainstem hemorrhage after discharge due to unmonitored anticoagulation. The bleeding issues are inevitably associated with the risk of thromboembolic events. Therefore, considering individual differences, careful adjustment of anticoagulant and antiplatelet therapy appears to be the only approach in treating this complication. According to guidelines, we used heparin bridging to warfarin for anticoagulation in the early postoperative period, with a target INR of 2.0–2.5 (25). Once the platelet count exceeded 100 × 10^9^/L, oral aspirin (100 mg/day) was initiated (10). Patients generally reached the target INR level on the third postoperative day. The cases of postoperative bleeding suggest that further exploration and optimization of the intensity of anticoagulation therapy are still needed.

Left ventricular failure is often accompanied by a certain degree of right ventricular dysfunction, and LVAD implantation may further increase the burden on the right heart, thereby elevating the risk of right heart failure (26). In this study, none of the patients developed right heart failure after surgery. This was attributed to the comprehensive preoperative assessment of the patients' right heart function and the postoperative adjustment of the patients' right heart function through medications, including intraoperative inhalation of nitric oxide and pulmonary vasodilator drugs support. In addition, for patients with moderate or severe TR preoperatively, we actively perform tricuspid valve repair during surgery to mitigate the adverse impact of tricuspid regurgitation on the right heart, which is crucial for preventing postoperative right heart failure. Moreover, an important experience in preventing postoperative right ventricular dysfunction is to perform transthoracic echocardiography examinations daily, focusing on the filling status of both the left and right ventricles. According to the ultrasound results, we timely adjust the LVAD speed and the doses of inotropic drugs or pulmonary vasodilators to reduce pulmonary vascular resistance, thereby minimizing left ventricular load, improving or maintaining right ventricular function, maintaining the correct position of the interventricular septum, keeping the degrees of aortic, mitral, and tricuspid regurgitation at the lowest possible levels, ensuring intermittent opening of the aortic valve, and maintaining adequate pump output and tissue perfusion.

Another aspect worthy of exploration in this study is the evaluation of organ function after CF-LVAD implantation in the early and late postoperative periods. Renal dysfunction is a relatively common complication after LVAD surgery and is closely associated with an increased postoperative mortality rate (7, 13, 27–29). It is currently widely believed that the main reasons for this phenomenon include alterations in renal perfusion, systemic oxygen supply imbalance, and triggering of an inflammatory cascade (30); additionally, nephrotoxic drugs used in the prevention of infections may also have adverse effects on renal function (31). Some studies have indicated that the period from 6 to 15 months postoperatively, the reduction in pulsatility caused by continuous blood flow does not have harmful effects on end-organ function (32, 33). In this study, most patients who underwent CF-LVAD implantation in the early postoperative period experienced elevated levels of creatinine. In addition to medications for protecting organ functions, five patients received CRRT for AKI. Encouragingly, the renal function of these patients had largely recovered when they were transferred out of the CICU. Moreover, during the 2-year follow-up period, the creatinine and blood urea nitrogen levels of these patients remained stable. Our study further confirms that the use of CF-LVAD may have a positive long-term effect on improving organ function, consisting with the finding of Wettersten et al.'s study (34). Nevertheless, as CF-LVAD technology advances and perioperative management develops, further long-term follow-up studies with large-sample studies are required to obtain more convincing data to verify the exact impact of CF-LVAD on organ function.

Given the particularity of this population, our center has established a follow-up management system led by cardiac surgeons as the core. This system implements comprehensive and detailed follow-up work for LVAD patients at different risk levels, fully considering factors such as cost-effectiveness and the risk of patient readmission (35). It comprehensively records and coordinates aspects such as pump operating parameter adjustments, general condition monitoring, infection monitoring, coagulation monitoring, organ function monitoring, 6 min walk tests, right heart catheterization and echocardiography examinations, patient psychological support, and education. Through this comprehensive, systematic, and personalized follow-up management system, our aim is to reduce patient readmission rates and achieve more favorable long-term survival rates for patients.

## Study strengths and limitations

The strengths of this study include uniform screening criteria, standardized surgical procedures, and consistent postoperative management strategies in all patients receiving CF-LVAD treatment. Additionally, this study incorporated comprehensive data from echocardiography, organ function, and long-term follow-up outcomes of CF-LVAD recipients.

However, this study still has some limitations. First, the retrospective design of this study can only reflect the management experience of a single center. With a small sample size and limited geographical representativeness, selection bias may exist. Expanding the sample size through multi-center collaboration is necessary to enhance the generalizability of the conclusions. Second, the study only included readmission data from our center and did not track cases treated at other hospitals, which may lead to an underestimation of the overall readmission rate. Third, due to the rapid emergence of various LVAD technologies, three different types of CF-LVAD devices were all included in this study. Due to the limited sample size and insufficient statistical power, we did not conduct a comparative analysis of the prognoses of patients assisted by different devices. Although all adopted magnetic levitation technology, differences in pump flow characteristics, anti-thrombotic coatings, and driveline designs may affect the homogeneity of adverse events. Therefore, larger-scale or more homogeneous patient cohorts (grouped by device type) are needed in the future to validate the long-term efficacy of CF-LVAD in treating ESHF. Forth, due to limited sample size and data volume, we did not delve deeply into the relationships between factors such as the length of device support, adverse events, medication adjustments, and blood pressure control, and clinical outcomes. Currently, our center is actively establishing a post-operative follow-up database for LVAD patients, which will encompass detailed information on support duration, adverse events, medication adjustments, and blood pressure control, among others. As the database continues to improve and the sample size gradually accumulates in the future, we will be able to conduct a detailed analysis of this information, thoroughly explore the relationships between various factors and clinical outcomes, and provide more valuable and comprehensive evidence for clinical practice. Finally, the significant improvement in ejection fraction observed after CF-LVAD implantation in this study has raised questions about the potential for myocardial recovery. Future studies should incorporate advanced imaging modalities to assess residual myocardial viability.

## Conclusion

Our center demonstrated a satisfactory survival rate exceeding three years that can be reasonably expected in patients undergoing CF-LVAD implantation through appropriate device implantation and patient management. However, considering the frequent readmissions in this population, patient survival rate, quality of life, and healthcare costs should be considered when interpreting these findings.

## Data Availability

The original contributions presented in the study are included in the article/Supplementary Material, further inquiries can be directed to the corresponding authors.
