# Supplementary material for: Perioperative clinical profiles and longitudinal outcomes in patients supported by continuous-flow left ventricular assist devices: a comprehensive analysis of early and late follow-up data
Source: Front Cardiovasc Med. 2026 Apr 14;13:1784700. doi: 10.3389/fcvm.2026.1784700 (PMC13121101; doi:10.3389/fcvm.2026.1784700)
Supplement: Supplementary file 1 [file Datasheet1.docx]

**Perioperative Clinical Profiles and Longitudinal Outcomes in Patients Supported by Continuous-Flow Left Ventricular Assist Devices:**

**A Comprehensive Analysis of Early and Late Follow-Up Data**

Qiuju Ding^#1^, Qingqing Zhu^#1^, Zeyi Zhou^#1^, Cheng Chen^1^, Zhenjun Xu^1^, Jun Pan*^1^, Min Ge*^1^

^1^Department of Cardio-thoracic Surgery, Nanjing Drum Tower Hospital, The Affiliated Hospital of Nanjing University Medical School, Nanjing, China.

**^#^**These authors contributed equally.

*Corresponding author:

Jun Pan, Ph.D., M.D.

Department of Cardio-thoracic Surgery, Nanjing Drum Tower Hospital, The Affiliated Hospital of Nanjing University Medical School, Zhongshan Road 321, Nanjing, China.

E-mail: [pj791028@163.com](mailto:pj791028@163.com)

Min Ge, Ph.D., M.D.

Department of Cardio-thoracic Surgery, Nanjing Drum Tower Hospital, The Affiliated Hospital of Nanjing University Medical School, Zhongshan Road 321, Nanjing, China.

Email: [gemin2000@outlook.com](mailto:gemin2000@outlook.com)

| **Supplementary Table S1.** Demographics, preoperative factors, and laboratory data of the patients on CF-LVAD support. | | |  |
| --- | --- | --- | --- |
| **Variable** | **Overall** | |  |
|  | **N = 27** | |  |
| Age, y, Median (IQR) | 61.00 (49.00, 69.00) | |  |
| **Age groups, n (%)** |  | |  |
| ≤45 y | 5 (18.5%) | |  |
| ＞45-≤60 y | 7 (25.9%) | |  |
| ＞60-≤80 y | 15 (55.6%) | |  |
| Gender, male, n (%) | 24 (88.9%) | |  |
| BMI, kg/m^2^, Mean ± SD | 24.57 ± 3.94 | |  |
| BMI<18.5 kg/m^2^, n (%) | 2 (7.4%) | |  |
| BMI≥30.0 kg/m², n (%) | 3 (11.1%) | |  |
| BSA in m^2^, Median (IQR) | 1.82 (1.67, 1.89) | |  |
| Smoking history, n (%) | 14 (51.9%) | |  |
| Drinking history, n (%) | 6 (22.2%) | |  |
| ICD, n (%) | 5 (18.5%) | |  |
| Reoperation, n (%) | 1 (3.7%) | |  |
| **Comorbidity, n (%)** |  | |  |
| Hypertension | 13 (48.1%) | |  |
| Diabetes mellitus | 15 (55.6%) | |  |
| CHD | 15 (55.6%) | |  |
| Prior PCI | 4 (14.8%) | |  |
| CKD | 3 (11.1%) | |  |
| Neurological dysfunction | 4 (14.8%) | |  |
| Atrial arrhythmia | 8 (29.6%) | |  |
| Ventricular arrhythmia | 3 (11.1%) | |  |
| **Cause of heart failure, n (%)** |  | |  |
| Idiopathic cardiomyopathy | 18 (66.7%) | |  |
| Ischemic cardiomyopathy | 9 (33.3%) | |  |
| **Pre-operative hemodynamic support** | | |  |
| ECMO support, n (%) | 1 (3.7%) | |  |
| On inotropic medicine, n (%) | 6 (22.2%) | |  |
| NYHA IV, n (%) | 13 (48.1%) | |  |
| INTERMACS 1-4, n (%) | 21 (77.8%) | |  |
| **Right heart catheterization** |  | |  |
| Systolic blood pressure (mmHg), Mean ± SD | 108.67 ± 14.30 | |  |
| Mean PAP (mmHg), Mean ± SD | 31.81 ± 13.40 | |  |
| PCWP (mmHg), Mean ± SD | 23.25 ± 10.01 | |  |
| CVP (mmHg), Mean ± SD | 7.67 ± 3.87 | |  |
| CO (L/min), Mean ± SD | 4.48 ± 1.58 | |  |
| Cardiac index (L/min/m^2^), Mean ± SD | 2.71 ± 1.04 | |  |
| PVR (Wood units), Median (IQR) | 2.13 (1.18, 2.40) | |  |
| **Serum biology** |  | |  |
| Sodium (mmol/L), Median (IQR) | 139.90 (137.80, 141.70) | |  |
| BUN (mmol/L), Mean ± SD | 10.84 ± 5.70 | |  |
| Creatinine (umol/L), Mean ± SD | 116.41 ± 83.37 | |  |
| Cys-C, mg/L, Median (IQR) | 1.26 (0.82, 2.24) | |  |
| eGFR, Mean ± SD | 73.17 ± 33.71 | |  |
| Albumin (g/L), Mean ± SD | 38.50 ± 4.92 | |  |
| ALT (U/L), Mean ± SD | 47.25 ± 68.41 | |  |
| AST (U/L), Mean ± SD | 34.88 ± 31.41 | |  |
| Total bilirubin (umol/L), Mean ± SD | 24.81 ± 21.05 | |  |
| Direct bilirubin (umol/L), Median (IQR) | 4.80 (2.40, 6.90) | |  |
| WBC (*10^9/L), Mean ± SD | 7.77 ± 2.76 | |  |
| Hemoglobin (g/L), Mean ± SD | 139.26 ± 23.73 | |  |
| Platelets (*10^9/L), Median (IQR) | 177.00 (134.00, 233.00) | |  |
| NT-proBNP (ng/L), Median (IQR) | 892.00 (615.00, 972.00) | |  |
| BNP (ng/L), Median (IQR) | 3,603.00 (2,698.00, 6,500.00) | |  |
| INR, Median (IQR) | 1.04 (0.98, 1.17) | |  |
| **Echocardiography** | 6 (22.2%) | |  |
| Ejection fraction (%), Mean ± SD | 25.81±5.54 | |  |
| LVDd (cm), Mean ± SD | 7.24 ± 0.78 | |  |
| LAD (cm), Mean ± SD | 4.88 ± 0.80 | |  |
| TAPSE, Mean ± SD | 1.57 ± 0.28 | |  |
| S' (cm/s), Mean ± SD | 35.25 ± 6.55 | |  |
| FAC (%), Median (IQR) | 8.50 (7.30, 10.00) | |  |
| Significant AR, n (%) | 1 (3.7%) | |  |
| Significant MR, n (%) | 12 (44.4%) | |  |
| Significant TR, n (%) | 1 (3.7%) | |  |
| **Medical treatment, n (%)** |  | |  |
| Beta-Blockers | 18 (66.7%) | |  |
| MRA | 18 (66.7%) | |  |
| SGLT2i | 10 (37.0%) | |  |
| ARNI | 8 (29.6%) | |  |
| Vericiguat | 12 (44.4%) | |  |
| **Abbreviation:** ALT, Alanine Aminotransferase; ARNI, Angiotensin Receptor-Neprilysin Inhibitor; AR, Aortic Regurgitation; AST, Aspartate Aminotransferase; BMI, body mass index; BNP, Brain Natriuretic Peptide; BSA, Body Surface Area; BUN, blood urea nitrogen; CHD, Coronary Heart Disease; CKD, chronic kidney disease; CO, Cardiac Output; CRRT, continuous renal replacement therapy; CVP, central venous pressure; Cys-C, cystatin C; ECMO, extracorporeal membrane oxygenation; FAC, Fractional Area Change; ICD, implantable cardioverter-defibrillator; INTERMACS, Interagency Registry for Mechanically Assisted Circulatory Support; LAD, Left Atrium Diameter; LVDd, Left Ventricular End Diastolic Diameter; MR, Mitral Regurgitation; MRA, Mineralocorticoid Receptor Antagonists; NYHA, New York Heart Association; PAWP, pulmonary arterial wedge pressure; PAP, Pulmonary Arterial Pressure; PCI, Percutaneous Coronary Intervention; PVR, Pulmonary Vascular Resistance; S', Systolic Peak Velocity; SGLT2i, Sodium-Glucose Co-Transporter 2 Inhibitors; TAPSE, Tricuspid Annular Plane Systolic Excursion; TR, Tricuspid Regurgitation; WBC, White Blood Cell. | | |  |
|  |  |  |  |
|  |  |  |  |
|  |  |  |  |
| **Supplementary Table S2.** Interoperative and postoperative factors of the patients on CF-LVAD support. | |  |  |
| **Variable** | **Overall** |  |  |
|  | **N = 27** |  |  |
| **Types of VADs, n (%)** |  |  |  |
| EVAHEART | 2 (7.4%) |  |  |
| HeartCon | 5 (18.5%) |  |  |
| Corheart | 20 (74.1%) |  |  |
| **Intraoperative condition, n (%)** |  |  |  |
| Tricuspid valve repair | 12 (44.4%) |  |  |
| Aortic valve replacement | 5 (18.5%) |  |  |
| Mitral valve repair | 3 (11.1%) |  |  |
| CABG | 6 (22.2%) |  |  |
| PFO closure | 4 (14.8%) |  |  |
| Concomitant RVAD insertion | 1 (3.7%) |  |  |
| Left atrial appendage resection | 6 (22.2%) |  |  |
| Duration of surgery, min, Median (IQR) | 325.00 (300.00, 409.00) |  |  |
| CPB duration, min, Mean ± SD | 157.31 ± 30.87 |  |  |
| Aortic clamping time, Mean ± SD | 106.73 ± 23.80 |  |  |
| Blood loss, mL, Median (IQR) | 1,200.00 (900.00, 1,400.00) |  |  |
| Blood transfusion, mL, Mean ± SD | 1,380.63 ± 618.84 |  |  |
| **Postoperative condition** |  |  |  |
| Chamber speed, rpm, Median (IQR) | 2,599.00 (2,498.00, 2,797.00) |  |  |
| Internet traffic, LPM, Mean ± SD | 3.98 ± 0.80 |  |  |
| Vasoactive drug VIS score, Median (IQR) | 53.00 (33.00, 85.00) |  |  |
| MAP, mmHg, Mean ± SD | 78.33 ± 10.02 |  |  |
| CVP, Median (IQR) | 13.00 (12.00, 16.00) |  |  |
| PAP, Median (IQR) | 20.50 (17.00, 25.00) |  |  |
| PAWP, Median (IQR) | 15.00 (11.00, 18.00) |  |  |
| LV/RV, Mean ± SD | 1.74 ± 0.16 |  |  |
| LA/RA, Median (IQR) | 1.32 (1.26, 1.37) |  |  |
| IVC, mm, Mean ± SD | 21.30 ± 1.45 |  |  |
| Variability of IVC, %, Median (IQR) | 9.00 (8.00, 15.00) |  |  |
| Balance D1, mL, Mean ± SD | -1,586.80 ± 852.86 |  |  |
| Balance D2, mL, Median (IQR) | -1,284.00 (-1,997.00, -339.60) |  |  |
| Balance D3, mL, Mean ± SD | -1,415.31 ± 1,005.47 |  |  |
| Duration of vasoactive drug use, day, Median (IQR) | 5.00 (3.00, 7.00) |  |  |
| Duration of mechanical ventilation, hours, Median (IQR) | 17.77 (14.67, 20.30) |  |  |
| CICU stay, day, Median (IQR) | 7.00 (5.00, 13.00) |  |  |
| Hospital stay, day, Median (IQR) | 48.00 (39.00, 56.00) |  |  |
| **Abbreviation:** CABG, coronary artery bypass graft; CPB, cardiopulmonary bypass; CVP, central venous pressure; IVC, inferior vena cava; LA, left atrium; LV, left ventricle; MAP, mean arterial pressure; PAWP, pulmonary arterial wedge pressure; PFO, Patent foramen ovale; RA, right atrium; RVAD, right ventricular assist device; RV, right ventricle. | |  |  |
|  |  |  |  |
|  |  |  |  |
|  |  |  |  |
